# Important Notice

**Published:** 1905-10

**Authors:** 


IMPORTANT NOTICE.—Judging from communications recently received, our reference to the "stegomyia fasciata" in connection with the "stegomyia punctata" has caused some physicians to suppose that we recommended Tongaline for yellow fever. This we emphatically disclaim.

The mention of these two species of mosquitoes was for the purpose of indicating that the mode of inoculation of yellow fever and malaria was precisely the same, and a careful reading of our statement will show that we had no intention to suggest that Tongaline was indicated in yellow fever, but on account of its pronounced eliminative action it did possess decided therapeutic value in the treatment of malaria.

We regret exceedingly that the notice referred to should have been misunderstood or misconstrued by anybody. MELLIER DRUG COMPANY, St. Louis.

(The reading notice referred to appeared only in medical journals.—Ed.)

